# Development and validation of a machine learning-based predictive model for assessing the 90-day prognostic outcome of patients with spontaneous intracerebral hemorrhage

**DOI:** 10.1186/s12967-024-04896-3

**Published:** 2024-03-04

**Authors:** Zhi Geng, Chaoyi Yang, Ziye Zhao, Yibing Yan, Tao Guo, Chaofan Liu, Aimei Wu, Xingqi Wu, Ling Wei, Yanghua Tian, Panpan Hu, Kai Wang

**Affiliations:** 1 Department of Neurology, The First Affiliated Hospital of Anhui Medical University, Hefei, China; 2 Anhui Province Key Laboratory of Cognition and Neuropsychiatric Disorders, Hefei, China; 3 Collaborative Innovation Centre of Neuropsychiatric Disorder and Mental Health, Hefei, 230000 China; 4 Center for Biomedical Imaging, University of Science and Technology of China, Hefei, 230026 Anhui China; 5 Department of Neurology, The Second People’s Hospital of Hefei, Hefei Hospital Affiliated to Anhui Medical University, Hefei, China; 6 Institute of Artificial Intelligence, Hefei Comprehensive National Science Center, Hefei, China; 7 Anhui Provincial Institute of Translational Medicine, Anhui Medical University, Hefei, China; 8 Department of Sleep Psychology, The Second Hospital of Anhui Medical University, Anhui Medical University, Hefei, China

**Keywords:** Spontaneous intracerebral hemorrhage, Prognosis, Prediction model, Machine learning

## Abstract

**Background:**

Spontaneous intracerebral hemorrhage (sICH) is associated with significant mortality and morbidity. Predicting the prognosis of patients with sICH remains an important issue, which significantly affects treatment decisions. Utilizing readily available clinical parameters to anticipate the unfavorable prognosis of sICH patients holds notable clinical significance. This study employs five machine learning algorithms to establish a practical platform for the prediction of short-term prognostic outcomes in individuals afflicted with sICH.

**Methods:**

Within the framework of this retrospective analysis, the model underwent training utilizing data gleaned from 413 cases from the training center, with subsequent validation employing data from external validation center. Comprehensive clinical information, laboratory analysis results, and imaging features pertaining to sICH patients were harnessed as training features for machine learning. We developed and validated the model efficacy using all the selected features of the patients using five models: Support Vector Machine (SVM), Logistic Regression (LR), Random Forest (RF), XGboost and LightGBM, respectively. The process of Recursive Feature Elimination (RFE) was executed for optimal feature screening. An internal five-fold cross-validation was employed to pinpoint the most suitable hyperparameters for the model, while an external five-fold cross-validation was implemented to discern the machine learning model demonstrating the superior average performance. Finally, the machine learning model with the best average performance is selected as our final model while using it for external validation. Evaluation of the machine learning model’s performance was comprehensively conducted through the utilization of the ROC curve, accuracy, and other relevant indicators. The SHAP diagram was utilized to elucidate the variable importance within the model, culminating in the amalgamation of the above metrics to discern the most succinct features and establish a practical prognostic prediction platform.

**Results:**

A total of 413 patients with sICH patients were collected in the training center, of which 180 were patients with poor prognosis. A total of 74 patients with sICH were collected in the external validation center, of which 26 were patients with poor prognosis. Within the training set, the test set AUC values for SVM, LR, RF, XGBoost, and LightGBM models were recorded as 0.87, 0.896, 0.916, 0.885, and 0.912, respectively. The best average performance of the machine learning models in the training set was the RF model (average AUC: 0.906 ± 0.029, P < 0.01). The model still maintains a good performance in the external validation center, with an AUC of 0.817 (95% CI 0.705–0.928). Pertaining to feature importance for short-term prognostic attributes of sICH patients, the NIHSS score reigned supreme, succeeded by AST, Age, white blood cell, and hematoma volume, among others. In culmination, guided by the RF model’s variable importance weight and the model's ROC curve insights, the NIHSS score, AST, Age, white blood cell, and hematoma volume were integrated to forge a short-term prognostic prediction platform tailored for sICH patients.

**Conclusion:**

We constructed a prediction model based on the results of the RF model incorporating five clinically accessible predictors with reliable predictive efficacy for the short-term prognosis of sICH patients. Meanwhile, the performance of the external validation set was also more stable, which can be used for accurate prediction of short-term prognosis of sICH patients.

## Introduction

Spontaneous intracerebral hemorrhage (sICH) emanates from the unheralded rupture of cerebral arteries, veins, and capillaries of diverse dimensions, absent any traumatic influence [1]. sICH is one of the most disabling and deadly subtypes of stroke, accounting for approximately 10% to 20% of all stroke types and is the second leading cause of death in the world population [2]. Therefore, it is particularly important to predict the prognosis and early intervention of patients with sICH. Extant research delineates a heightened sICH incidence in middle- and low-income nations compared to their affluent counterparts (117 per 100 000 and 94 per 100 000, respectively), with a notable global surge in Asia relative to other ethnic contingencies [3].The disease burden of sICH is largely attributable to neglect of the management of controllable risk factors[4, 5]. Consequently, establishing a practical risk prediction model for sICH prognosis is crucial, enabling enhanced, precise management and improved patient outcomes.

Machine Learning (ML) has evolved into a potent computer-assisted method for data mining and analysis, garnering extensive application as a predictive instrument across diverse engineering and medical contexts [6, 7]. The predictive accuracy of ML proves superior to that of conventional statistical approaches [8, 9]. Previous studies indicate the linkage of patients with sICH prognosis to numerous elements, including demographic factors, hematoma volume, site of hemorrhage, inflammatory responses, and pharmaceutical impacts, cumulatively influencing the prognosis outcomes of sICH patients [10–13]. ML can analyze extensive data sets to uncover obscured predictive risk factors for enhanced clinical direction [14]. Previous studies have developed some predictive models for the short-term prognosis of patients with sICH using an imaging histology approach, which has high predictive efficacy but is not amenable to clinical practice [15, 16]. Furthermore, numerous studies fail to conduct external validation of their models, thereby constraining the model's generalizability and its broader applicability [17, 18]. Meanwhile, the vast majority of studies have only conducted model training and testing for machine learning by dividing the dataset randomly at one time, which increases the selection bias of the dataset and ultimately leads to unstable or even inaccurate performance of the model [19]. Other studies, despite constructing predictive models, have not grounded them in a platform for feasible use, thereby curtailing their clinical utility [20, 21].

Therefore, our research endeavors to contrast diverse machine learning models to predict the short-term prognostic outcome for sICH patients, based on various clinical features. Our objective further extends to ascertaining the significance of disparate features in influencing the prognostic outcome of sICH. Ultimately, we establish a simplified and efficient prediction platform founded on the most efficacious machine learning model, enhancing clinical practice value.

## Methods and materials

### Patients

We retrospectively collected 413 consented sICH patients admitted in the Department of Neurology at the Second People's Hospital of Hefei from January 2018 to March 2022 and included them in the study sample. For the validation sample, 74 cases of consented sICH patients admitted in the Department of Neurology at the First Affiliated Hospital of Anhui Medical University from December 2022 to May 2023 were composed for training, validating and testing the machine learning model. We included the patients with the following criteria: (1) age > 18 years; (2) meeting the diagnostic criteria for sICH established by the Cerebrovascular Disease Group of the Chinese Academy of Neurology, and the diagnosis was consistent with cranial CT scan; (3) time between the onset and first cranial CT examination < 24 h. Secondary cerebral hemorrhage, such as trauma, cerebral infarction with cerebral hemorrhage transformation, cerebrovascular malformation, and brain tumor, was also excluded. Exclude subarachnoid hemorrhage. Exclude patients who have undergone surgery, intervention, or other surgical instrumentation prior to the review of cranial CT. Exclude patients who have lost visits after sICH.

This study was approved by the Research Ethics Committees of the Hospital of Hefei Affiliated with Anhui Medical University (2023-yan-018) and the First Affiliated Hospital of Anhui Medical University (2021H048). All participants or their guardians agreed to the study and signed the informed consent forms.

### Data acquisition

We collected characteristics such as general demographic characteristics, past medical history, laboratory tests and general imaging data of the patients. 1. demographic characteristics, including age, sex, smoking and drinking; 2. past medical history, including history of hypertension, diabetes mellitus; 3. laboratory tests, including white blood cells, neutrophils, lymphocyte and so on; 4. general imaging, including hematoma volume, bleeding location and so on.

### Statistical methods

#### Selection of candidate variables and predictors

This study encompasses clinically pertinent characteristics of sICH patients, gathered at the point of admission. The attributes under consideration entail various dimensions, including general demographic characteristics, past medical history, laboratory tests and general imaging data. Continuous variables underwent standardized processing, while categorical variables were addressed with one-hot encoding methods.

Employing Recursive Feature Elimination (RFE), the study sifted for the superior subset to procure the most favorable combination of features. RFE is a mainstream screening method for machine learning feature screening. RFE removes features that are not important for the ending variables, and ultimately obtains the optimal combination of variables for the best performance of the model [22]. RFE helps to improve the performance of predictive models, especially in avoiding overfitting, and is beneficial in improving the generalization ability of the model. RFE reduces the number of features and makes the model simpler, easier to interpret, which is beneficial for clinical applications [23]. Following this, the refined optimal subset feature tables were integrated into our quintet of machine learning models for concurrent training and testing.

#### Machine learning models

In this study, five distinct machine learning models were employed for both training and testing, namely, Support Vector Machines (SVM), Logistic Regression (LR), Random Forest (RF), LightGBM, and XGBoost.

SVM is a supervised machine learning algorithm that can be used for regression and classification problems. It functions by delineating data into decision boundaries for varied classes, concurrently maximizing the margin between these boundaries and the nearest data instances, thereby enhancing the model’s classification performance and generalization capability.

LR is a generalized linear regression model which is commonly used to solve classification problems, this model is easy to understand and explain.

RF is an integrated learning method based on decision trees. It operates on the logic of improving the accuracy and robustness of the model by constructing multiple decision trees based on random samples and random features. This model is a powerful machine learning model and is a good choice for solving classification problems.

LightGBM is a high-performance gradient boosting decision tree based running framework commonly used to solve classification and regression problems. Its unique histogram gradient boosting method and leaf-wise learning strategy make it perform well in large datasets, and it is a powerful tool for solving classification problems.

XGboost is a mainstream machine learning model. It is an integrated learning method based on gradient boosting tree, which further improves the accuracy of the model by constructing multiple decision trees to reduce the prediction error. At the same time, the model can support multiple loss functions and regularization features, making XGboost known for its high performance and scalability in the field of machine learning.

In this study, every enlisted machine learning model was meticulously configured to utilize the aforementioned scrutinized features, with the aim to effectively differentiate between poor and good short-term prognoses for sICH patients.

#### Selection of machine learning models

The dataset within the training set is divided in a 7:3 ratio. This division allocated seven parts for model training, utilizing the remaining three parts for testing model performance. Internal fivefold cross-validation was employed to discern the most suitable hyperparameters for each distinct model, individually applied to each model for enhanced precision. Moreover, external fivefold cross-validation facilitated the comparison of machine learning models, identifying the model with superior average performance as the ultimate predictive model.

Evaluation metrics, including AUC and accuracy, served to assess each model’s performance. The SHAP method was employed to showcase the important weight of each variable, offering insights into their relative importance within the model. In conclusion, the optimal amalgamation of predictor variables was determined by integrating variable importance weight and combinations, culminating in the establishment of a comprehensive prediction platform.

#### Additional statistical techniques

Data analysis and visualization were conducted utilizing SPSS (version 24.0), Python (version 3.10.10), Scikit-learn (version 1.2.2) and Shiny (version 0.5.1). Categorical variables underwent evaluation with chi-square or Fisher’s test, with the findings outlined in percentage terms. Continuous variables adhering to normal distribution were depicted as mean ± standard deviation, and scrutinized using the t-test. Non-normally distributed data were characterized using quartiles and assessed with non-parametric tests. A p-value under 0.05 (two-tailed) was considered indicative of statistical significance.

## Results

### Clinical characteristics

Table 1 provides a comparison of the baseline characteristic between the training set and external testing set data. No substantial differences were observed between the training set and the external test set across the majority of features. The proportion of patients with hypertension was notably higher in the external test set than in the training set (91.89% VS 72.64%, P < 0.001). Contrarily, the proportion of patients consuming alcohol was significantly elevated in the training set compared to the external test set (28.57% VS 13.51%, P = 0.007). Concurrently, the incidence of intraventricular hemorrhage was appreciably higher in the training set than in the external test set (24.32% VS 9.2%, P < 0.001). Moreover, uric acid levels were markedly higher in the training set (351.04 ± 123.55 VS 266.43 ± 105.84, P = 0.00). In the external test set, the AST was significantly elevated compared to the training set (32.31 ± 21.13 VS 26.42 ± 12.17, P = 0.022). Furthermore, in the training set, GCS scores were significantly lower (12.86 ± 3.42 VS 13.70 ± 3.08, P = 0.047), while NIHSS scores were considerably higher (10.08 ± 9.73 VS 7.59 ± 8.56, P = 0.04) (Table 1).

**Table 1:** Demographics and clinical characteristics of study in the training and validation cohorts

Group	All data	Train data	Test data	P-value
N	487	413	74	
Age				0.460
< 65	322 (66.12%)	209 (50.61%)	34 (45.95%)	
> = 65	165 (33.88%)	204 (49.39%)	40 (54.05%)	
Sex				0.176
Male	322 (66.12%)	268 (64.89%)	54 (72.97%)	
Female	165 (33.88%)	145 (35.11%)	20 (27.03%)	
Hypertension				< 0.001
No	119 (24.44%)	113 (27.36%)	6 (8.11%)	
Yes	368 (75.56%)	300 (72.64%)	68 (91.89%)	
Diabetes				0.942
No	429 (88.09%)	364 (88.14%)	65 (87.84%)	
Yes	58 (11.91%)	49 (11.86%)	9 (12.16%)	
Smoking				0.071
No	375 (77.00%)	312 (75.54%)	63 (85.14%)	
Yes	112 (23.00%)	101 (24.46%)	11 (14.86%)	
Drinking				0.007
No	359 (73.72%)	295 (71.43%)	64 (86.49%)	
Yes	128 (26.28%)	118 (28.57%)	10 (13.51%)	
Hematoma volume				0.160
< 20	383 (78.64%)	322 (77.97%)	61 (82.43%)	
20–40	55 (11.29%)	45 (10.90%)	10 (13.51%)	
> = 40	49 (10.06%)	46 (11.14%)	3 (4.05%)	
Intraventricular hemorrhage				0.559
No	355 (72.90%)	299 (72.40%)	56 (75.68%)	
Yes	132 (27.10%)	114 (27.60%)	18 (24.32%)	
Infratentorial hemorrhage				< 0.001
No	431 (88.50%)	375 (90.80%)	56 (75.68%)	
Yes	56 (11.50%)	38 (9.20%)	18 (24.32%)	
Outcome				0.176
Good outcome	281 (57.70%)	233 (56.42%)	48 (64.86%)	
Poor outcome	206 (42.30%)	180 (43.58%)	26 (35.14%)	
White blood cell	8.65 ± 3.80	8.69 ± 3.93	8.42 ± 2.98	0.577
Neutrophils	6.86 ± 4.87	6.72 ± 3.77	7.62 ± 8.79	0.389
Lymphocyte	1.39 ± 0.69	1.41 ± 0.69	1.33 ± 0.69	0.431
Urine nitrogen	5.73 ± 2.69	5.76 ± 2.74	5.59 ± 2.46	0.63
Creatinine	79.93 ± 54.24	80.70 ± 54.16	75.61 ± 54.85	0.458
Uric acid	338.18 ± 124.69	351.04 ± 123.55	266.43 ± 105.84	0.00
ALT	21.72 ± 11.64	21.37 ± 11.32	23.71 ± 13.22	0.155
AST	27.32 ± 14.04	26.42 ± 12.17	32.31 ± 21.13	0.022
Glucose	6.70 ± 2.45	6.69 ± 2.49	6.73 ± 2.20	0.925
Systolic pressure	162.71 ± 26.85	163.69 ± 27.29	157.23 ± 23.68	0.057
Diastolic pressure	93.31 ± 17.30	93.90 ± 17.30	89.99 ± 17.04	0.073
GCS scores	12.99 ± 3.38	12.86 ± 3.42	13.70 ± 3.08	0.047
NIHSS scores	9.70 ± 9.59	10.08 ± 9.73	7.59 ± 8.56	0.040

Table 2 delineates the disparities between groups regarding the varied prognoses of sICH patients in the training set. Within the poor prognosis group, the ratio of elderly patients was significantly augmented compared to the good prognosis group (56.1% VS 44.2%, P = 0.016). The incidence of intraventricular hemorrhage was markedly elevated in the poor prognosis group compared to the good prognosis group (48.9% VS 11.2%, P = 0.000). Additionally, a significant distinction in hematoma volume was noted between the two groups (P = 0.000) (Table 2).

**Table 2:** ICH patients’ characteristics in the Training cohort

Characteristic	Good outcome	Poor outcome	P-value
Age			0.016
< 65	130(55.8%)	79(43.9%)	
> = 65	103(44.2%)	101(56.1%)	
Sex			0.228
Male	157(67.4%)	111(61.7%)	
Female	76(32.6%)	69(38.3%)	
Hypertension			0.867
No	63(27.0%)	50(27.8%)	
Yes	170(73.0%)	130(72.2%)	
Diabetes			0.303
No	31(13.3%)	162(90.0%)	
Yes	202(86.7%)	18(10.0%)	
Smoking			0.105
No	169(72.5%)	143(79.4%)	
Yes	64(27.5%)	37(20.6%)	
Drinking			0.233
No	161(69.1%)	134(74.4%)	
Yes	72(30.9%)	46(25.6%)	
Intraventricular hemorrhage			0.000
No	207(88.8%)	92(51.1%)	
Yes	26(11.2%)	88(48.9%)	
Infratentorial hemorrhage			0.379
No	209(89.7%)	166(92.2%)	
Yes	24(10.3%)	14(7.8%)	
Hematoma volume			0.0000
< 20 ml	217(93.1%)	105(58.3%)	
20–40 ml	14(6.0%)	31(17.2%)	
> = 40 ml	2(0.9%)	44(24.4%)	
White blood cell	7.47 ± 2.26	10.27 ± 4.96	0.000
Neutrophils	5.46 ± 2.05	8.35 ± 4.74	0.000
Lymphocyte	1.47 ± 0.70	1.33 ± 0.68	0.043
Urine nitrogen	5.40 ± 2.22	6.21 ± 3.24	0.005
Creatinine	80.49 ± 64.55	80.96 ± 36.78	0.931
Uric acid	348.82 ± 113.15	353.91 ± 136.11	0.686
ALT	21.26 ± 12.17	21.50 ± 10.14	0.829
AST	24.13 ± 8.41	29.38 ± 15.28	0.000
Glucose	6.13 ± 1.86	7.43 ± 2.99	0.000
Systolic pressure	159.55 ± 25.37	169.04 ± 28.79	0.00
Diastolic pressure	92.66 ± 16.82	95.5 ± 17.82	0.099
GCS scores	14.53 ± 1.20	10.69 ± 4.07	0.000
NIHSS scores	4.60 ± 4.44	17.16 ± 10.13	0.000

### Selection of predictors

We employ a RFE strategy for feature screening. The amalgamation of optimal subsets ascertained according to the recursive feature elimination method includes: NIHSS score, AST, Age, White Blood Cell, Hematoma volume, Urine nitrogen, Neutrophils, Glucose, Creatinine, Systolic Pressure, ALT, Lymphocyte, Diastolic Pressure, Uric acid, GCS score.

### Multiple machine learning model performance

**Fig. 1:**
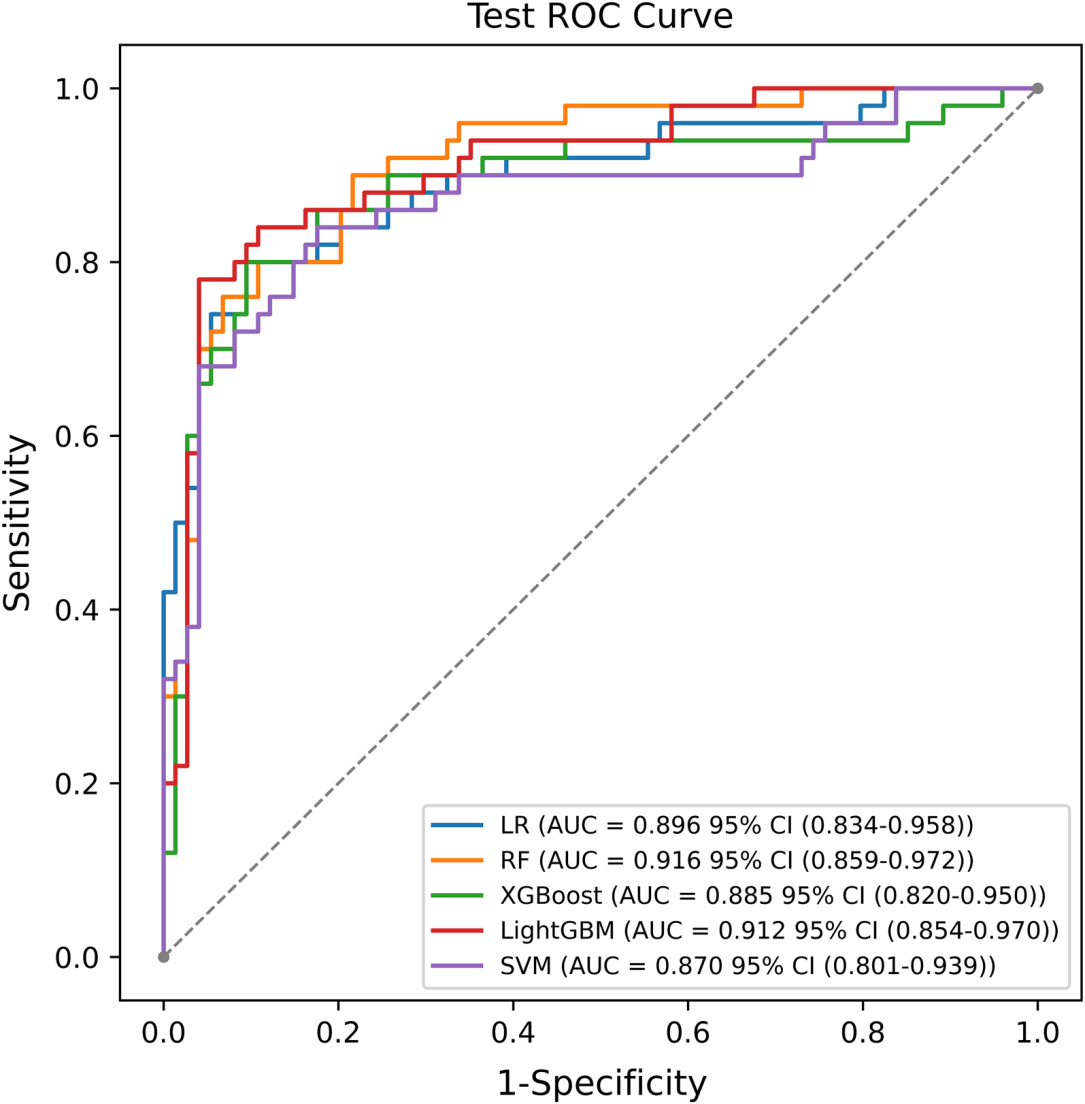
ROC curve analysis of the five machine learning alorithms for predicting short-term prognosis of ICH patients in the test data

**Fig. 2:**
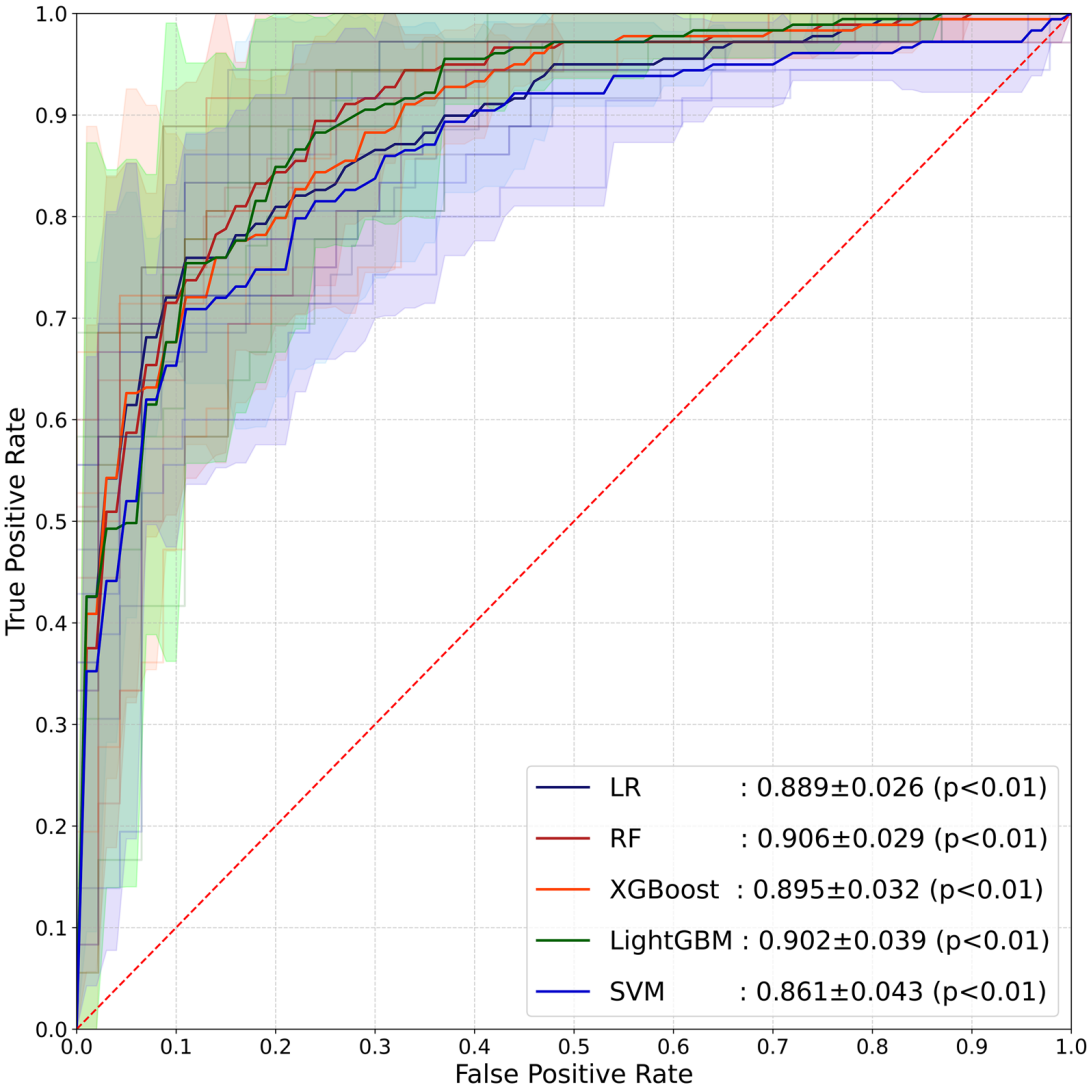
The average AUC performance of five machine learning models subjected to fivefold external cross-validation

We based our model training and testing on the aforementioned selected features. The AUC of all models on the internal test set ranged between 0.85 and 0.95, with the RF model emerging as the most efficacious [AUC: 0.916, 95% CI (0.859–0.972)] (Fig. 1). During the external fivefold cross-validation, the mean performance of the RF persistently ranked superior (AUC: 0.906 ± 0.029) (Fig. 2). Table 3 illustrates a comparison of common performance metrics among diverse machine learning prediction models. Based on these outcomes, we select the RF model as our concluding risk prediction model.

**Table 3:** Comparative analysis of the performance outcomes across various machine learning models

Model	F1 score (%)	Accuracy (%)	Recall (%)	Precision (%)	AUC (%)	Sensitivity (%)	Specificity (%)
LR model	80.8	84.7	80.0	81.6	89.6	80.0	87.8
RF model	78.5	81.5	84.0	73.7	91.6	84.0	79.7
XGBoost model	81.1	83.9	86.0	76.8	88.5	86.0	82.4
LightGBM model	79.6	82.3	86.0	74.1	91.2	86.0	79.7
SVM model	78.0	82.3	78.0	78.0	87.0	78.0	85.1

*LR* logistic regression; RF, random forest; XGBoost, extreme gradient boosting; *LightGBM* light gradient boosting machine; *SVM* support vector machine; *AUC* area under the curve

**Fig. 3:**
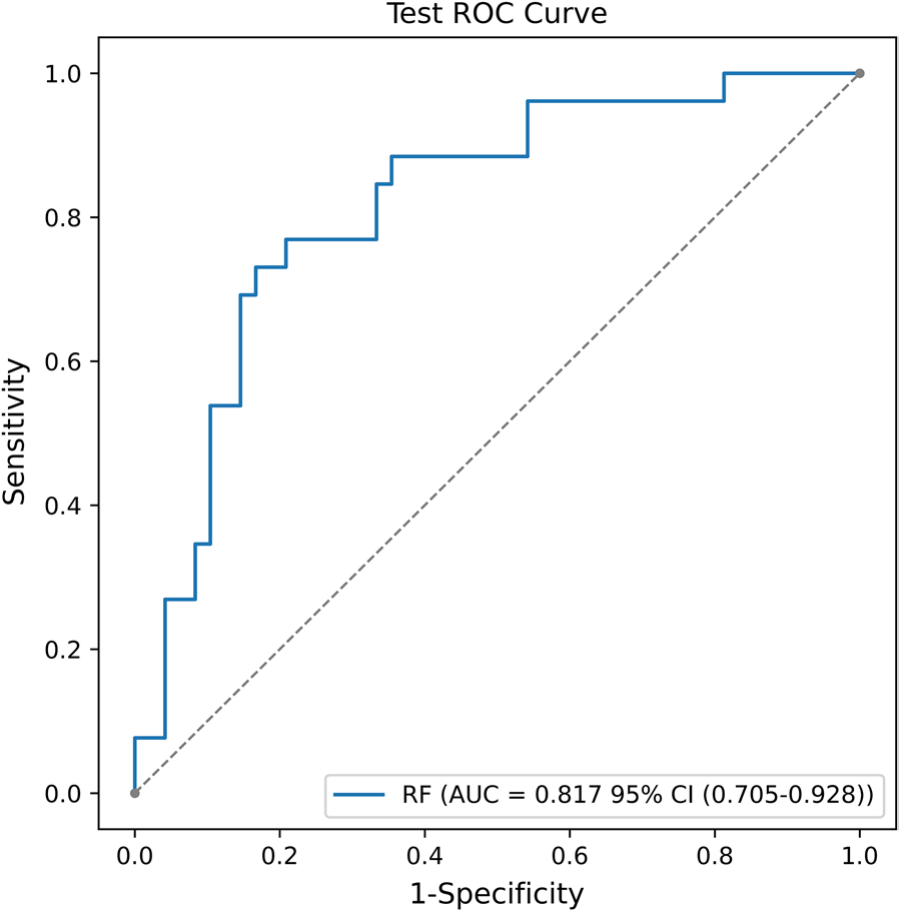
ROC curve analysis of the RF alorithms for predicting short-term prognosis of ICH patients in the external test set

The performance of the RF model, trained as described, remains stable in the external test set (AUC: 0.817, 95% CI (0.705–0.928)) (Fig. 3).

### Variable importance and variable interpretation

**Fig. 4:**
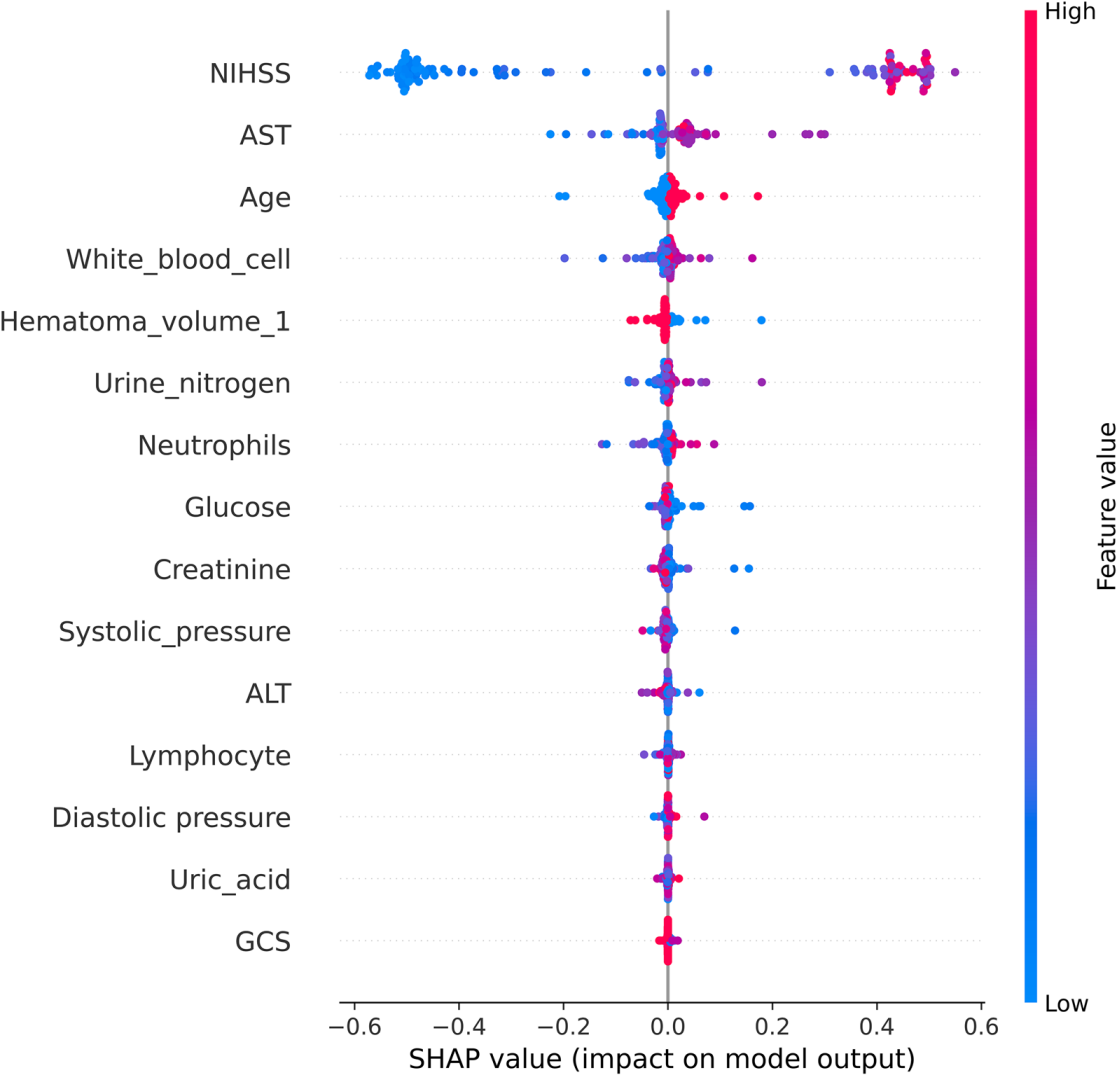
SHAP analyses of the RF model for predicting poor prognosis of ICH patients

We visualize the impact of predictor variables on outcomes based on SHAP plots. Specifically, the influence of a variable on the outcome can be visually interpreted through the magnitude of the SHAP value (indicated by a change in color) and the trend on the horizontal axis of the variable (probability of developing a poor outcome). For instance, in the scenario of NIHSS scores, individuals with elevated scores (represented in red) are more prone to have an adverse prognosis (on the right-hand side) compared to those with lower NIHSS scores (depicted in blue). Similarly, for individuals with augmented AST levels (in red), the prognosis for sICH patients is likely to be unfavorable (right side). The prognosis for patients with sICH may be unfavorable for those individuals whose hematoma volume is not hematoma volume_1 (hematoma volume > 20 ml, indicated in blue) (located on the right) (Fig. 4).

### Implementation of web calculator

**Fig. 5:**
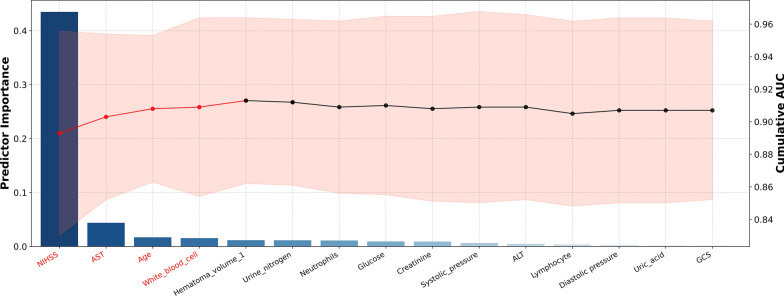
Comparison of the performance derived from RF model constructed with various variable combinations based on variable importance

**Fig. 6:**
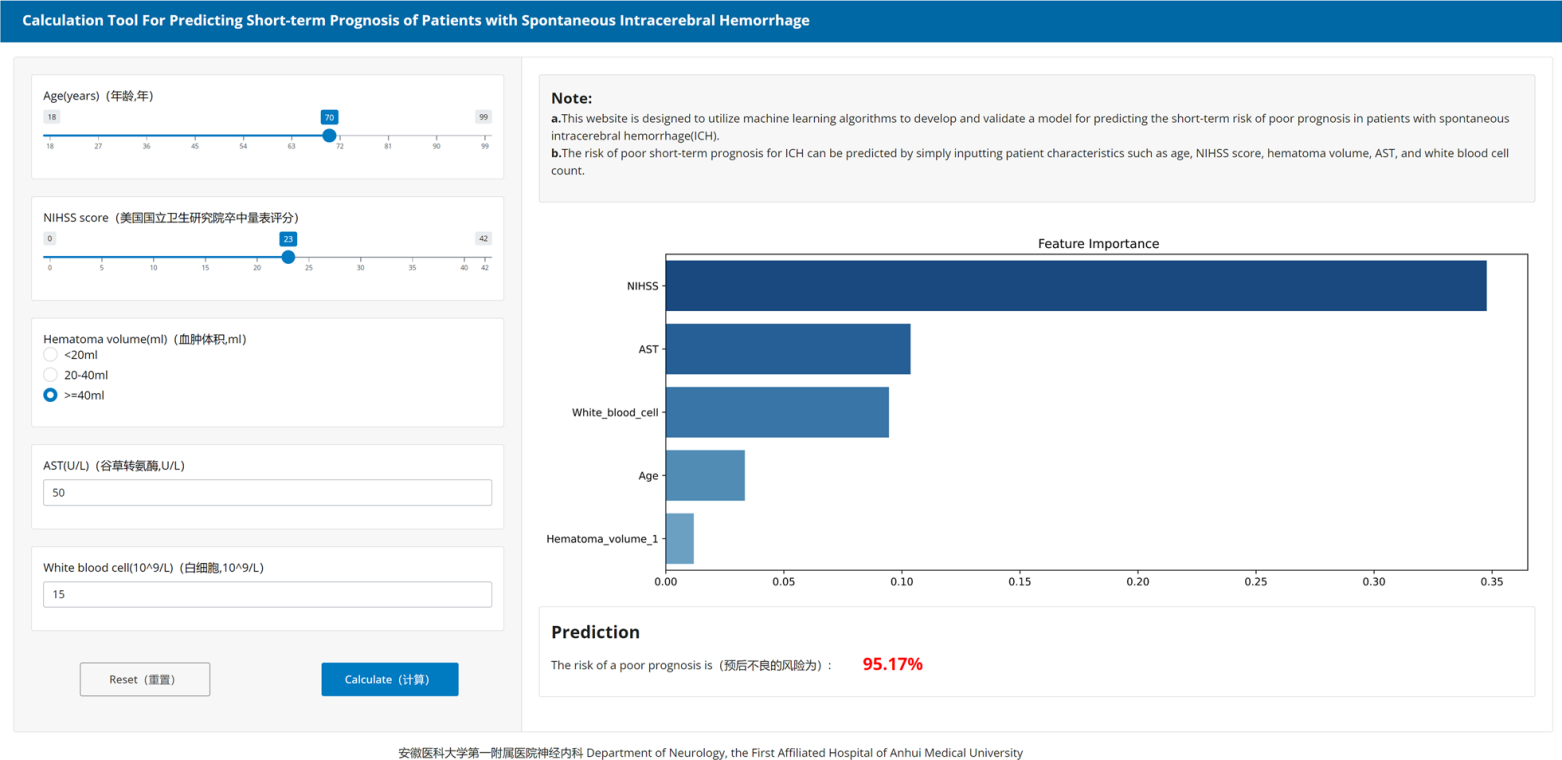
A web-based calculator for predicting short-term prognosis in patients with ICH

We additionally plotted the combined AUC and variable importance line graphs, utilizing model prediction data constituted by variable importance and variable combinations from RF model. As per the figure, it is evident that the amalgamation of variables including NIHSS score, AST level, Age, White blood cell, and Hematoma volume is capable of attaining the optimized and streamlined predictive efficacy (Fig. 5). A web calculator was constructed based on these five indicators, facilitating individualized prediction of prognostic risk in sICH patients (https://surge-ustc.shinyapps.io/hemorrhage_prognosis/) (Fig.  6).

## Discussion

The dangers of sICH cannot be underestimated. Research data indicates that the disability rate of sICH soars between 40 and 80%, and almost half of the affected individuals succumb within one-month post-onset of the ailment [24, 25]. When cerebral hemorrhage occurs, blood permeates into the brain parenchyma from a burst cerebral vessel, potentially escalating intracranial pressure and inflicting damage to adjacent brain cells [26]. This cascade can lead to pronounced neurological dysfunction. A severe cerebral hemorrhage may precipitate limb paralysis, aphasia, coma, and in dire circumstances, death [27]. Prognosticating the outcome of sICH enhances our understanding of patient conditions and potential risks, enabling the administration of more tailored therapeutic interventions. Solely considering the condition, numerous factors influence the prognosis of sICH, chiefly among them being the site and volume of bleeding. Nevertheless, the prognosis is not rigid, and factors such as patient age and preceding health status exert significant influence [28]. Consequently, even seasoned neurologists find it challenging to predict the short-term outcome of sICH. Therefore, establishing a systematic prediction platform for short-term prognosis of sICH patients and realizing online calculation of individual risks has important clinical practice value.

In this individual-level analysis of a retrospective study cohort, a newly devised machine-learning-based tool was developed for the prediction of short-term prognosis in patients with sICH. From a relatively large number of health- and prognostic-related variables, a series of data-driven selection approaches were utilized, and the five most pivotal predictors were identified. The RF model predicted the short-term prognosis in sICH with an AUC of 0.916, indicating a high predictive performance. Enhanced performance was also observed upon its application to the prediction of an external validation dataset. Our proposed clinical prognostic prediction tool is effortless to implement in clinical settings, enabling a swift prognosis of clinical outcomes, contributing significantly to clinical decision making.

Amidst the progression of machine learning, random forest models emerge as a superior methodology for constructing relevant medical predictive models. Previous studies by Huang et al. have shown that RF models can improve the prediction capability of prognosis in acute respiratory distress syndrome [29]. In the present study, the RF model identified NIHSS score, AST level, age, white blood cell counts and hematoma volume as the top 5 risk factors for short-term prognosis of sICH. This model uses the simplest combination of variables while achieving the best predictive performance. To reduce the risk of dataset selection bias due to random dataset splitting, we performed external fivefold cross-validation of all machine learning models to obtain the average performance of each machine learning model based on five predictions. Results from external cross-validation reveal that RF model (AUC: 0.906 ± 0.029) outperform other machine learning models in terms of average predictive performance. The findings indicate that the AUC of the RF model in the testing set stands at 0.916(95% CI 0.827–1.005), surpassing other models. Concurrently, within the external validation dataset, the AUC of the RF model reached 0.817, signaling the robust generalization capability of the RF model, affirming its applicability in clinically predicting sICH short-term prognosis.

The NIHSS score has garnered extensive utilization in clinical trials concerning acute ischemic stroke for the assessment of stroke severity [30]. A research endeavor spearheaded by UK academics sought to authenticate the association between NIHSS scale score items and prognosis in hyperacute-phase stroke patients undergoing thrombolysis treatment. The study outcomes unveiled a significant association between NIHSS score items, functional prognosis, and mortality in patients enduring acute ischemic stroke under thrombolysis [31]. In patients with hemorrhagic stroke, NIHSS scores have garnered escalating attention recently [32]. Our study echoes this by demonstrating a correlation between elevated NIHSS scores and a dismal short-term prognosis, aligning with prior research [21, 33]. Our study also found that patients with higher AST would have poorer prognosis. This is consistent with the findings of Tan et al. [34]. This may be due to the fact that AST is a glutamate-regulating enzyme, and higher AST levels lead to higher glutamate levels, and the neurotoxicity of glutamate leads to a poorer prognosis for patients [35, 36]. Moreover, our findings also indicate that age is a significant factor of poor prognoses in patients with sICH. The results show an increased risk of poor prognosis in sICH patients with advancing age. As patients age, their physiological reserves decline and they are less able to recover from a cerebral hemorrhage event. Previous studies have also revealed that the effects of ageing on hematoma volume and neuroinflammation exacerbate the poor prognosis of sICH patients [37, 38]. Our results also show that as white blood cell levels increase, the risk of poor prognosis in sICH patients also increases significantly. An augmented white blood cell count typically signifies the manifestation of an inflammatory response within the organism. sICH precipitates both localized and systemic inflammatory reactions, which, in the context of sICH, can induce collateral damage to the adjacent cerebral tissue and potentiate the severity of cerebral edema [39]. Concurrently, post-hemorrhagic immune cell aggregation, encompassing white blood cell, at the hemorrhage locus could amplify neuronal injury through the secretion of pro-inflammatory cytokines and proteolytic enzymes [40]. We also found that patients with smaller hematoma volumes (< 20 ml) had a better prognosis relative to those with larger hematoma volumes, which is consistent with many previous studies [41].

The strength of this study is the comparison of different ML models to predict the short-term prognosis of sICH. The external validation performance and comparison with other models also demonstrated that the RF model has a good predictive value for short-term prognosis of sICH. For further application, we built a user-friendly online prediction platform for neurologists and patients worldwide.

Certainly, our study has some limitations. Firstly, leukocyte counts may be influenced by a number of factors, such as the use of medication. Secondly, the present study is a retrospective paired-cohort study and there may be some bias in the results of the study. Furthermore, the limited number of cases in external validation centers may limit the reliability of the present results. Future research endeavors should engage in multi-center validation and embark on large-scale prospective studies to enhance the robustness of our findings.

In conclusion, a predictive model has been established, leveraging the outcomes of the RF model and integrating four clinically attainable predictors. This model exhibits dependable predictive efficacy for the short-term prognosis of sICH patients. Meanwhile, the performance of the external validation set was also more stable, which can be used for accurate prediction of short-term prognosis of sICH patients.

## Data Availability

The data used in this study are available from the corresponding author upon reasonable request.
